# Risk of HIV/STIs among Muslim army conscripts in the three deep southern provinces of Thailand

**DOI:** 10.34172/hpp.2021.56

**Published:** 2021-12-19

**Authors:** Awirut Singkun, Kraiwuth Kallawicha, Khemika Yamarat

**Affiliations:** College of Public Health Sciences, Chulalongkorn University, Bangkok, Thailand

**Keywords:** Human immunodeficiency virus, Islam, Military, Sexual behavior, Sexually transmitted diseases, Thailand

## Abstract

**Background:** The prevalence of sexually transmitted diseases is an important public health problem, especially in people who are sexually active, such as the army conscript group. However, their knowledge, attitudes, and practices may be influenced by certain cultures and beliefs. This study explored the factors associated with the risk of human immunodeficiency virus (HIV)/sexually transmitted infections (STIs) among the Muslim army conscript in three deep southern provinces of Thailand.

**Methods:** The cross-sectional study design was conducted among 360 Muslim army conscripts. A researcher made questionnaire on knowledge of HIV/STI transmission, attitude toward condom use, and sexual behavior was distributed to the participants in a camp base. The association between the potential predictor variables and the risk of HIV/STI was analyzed using the multiple logistic regression. Statistically significant of the association considered a *P* value ≤ 0.05.

**Results:** The results suggest that most Muslim army conscripts had a poor knowledge level of HIV/STI transmission (78.7%) and that their attitude toward condom use was at a moderate level (60.0%). The predicted factors were marital status [aOR=0.078, 95% CI=0.035-0.172], substance use before having sex [aOR=8.044, 95% CI=1.288-50.230], stimulant use before having sex [aOR=3.632, 95% CI=1.080-12.211], vaginal sexual intercourse [aOR=26.228, 95% CI=8.370-82.189], and oral sexual intercourse [aOR=2.256, 95% CI=1.106-4.601].

**Conclusion:** A proper sexual health education program should be developed and delivered to enhance knowledge on HIV/STI transmission among Muslim army conscripts.

## Introduction

Human immunodeficiency virus (HIV) and sexually transmitted infections (STIs) are sexually transmitted diseases that have caused a public health problem worldwide over the past decades. Not only are they a health burden among patients, but they are also a cause of social problems, social stigma, discrimination, and violation of human rights.^1^ In Thailand, the HIV prevalence rate was 20.5% among injection drug users, 11.9% among men who have sex with men, 11.0% among transgenders, 2.8% among sex workers, 1.1% among sexually active men, and 1.0% among adults overall.^2^

Among the vulnerable groups for HIV/STI, army conscripts are a high-risk group for infection.^3,4^ The prevalence rate of HIV among Thai army conscripts increased by approximately 0.5% from 2007 to 2013, and the trend is expected to increase.^4^ The latest report in 2016 indicated that the HIV prevalence rate among first-shift army conscripts was 0.78%.^1,4^ In the US Army, the prevalence rate of HIV/STI among 21-25-year-old male US Army soldiers was 4.2%, and 25.0% of them reported five or more sexual partners in the past year.^3^ Although the prevalence rate among Thai Army conscripts is lower, it is still an important problem that needs to be addressed.

A study on STI risk behaviors in young Thai adults found that unprotected sexual intercourse with casual partners was the greatest risk factor for contracting HIV and other STIs. A national survey among army conscripts of the Royal Thai Army reported that 90.0%–96.2% engaged in some form of a sexual act and had an average of six sexual partners in their lifetime.^5^ About 25.4% had sexual experience with female sex workers, 8.0% had sexual intercourse with men, and 7.3% had sex in exchange for something.^6^ Another study on army conscripts reported that more than half of the participants (58.8%) did not use condoms during their last sexual intercourse, 20.0% never used condoms, and 5.2% had 10–15 sexual partners within the past six months.^5^ Moreover, there have been reports of sexual abuse in military environments, with sex between males being an important risk behavior.^7^. Another study on the risk factors for HIV infection among Thai army conscripts found that smoking, alcohol consumption, substance addiction, and multiple sexual partners were the major risk factors.^8^ Thus, army conscripts are considered to have a higher risk of HIV and other STIs than the general population.^3,9^

Every year, in accordance with Thai military law, 21-year-old men are recruited through a lottery system to be enlisted army conscripts. They are predominantly young, single, and sexually active men.^6,8,10^ The three southernmost provinces of Thailand, namely, Yala, Pattani, and Narathiwat, have a higher proportion of army conscripts than other parts of Thailand due to unrest. The sectarian violence that has occurred since 2004 in this area has left approximately 5600 dead and 10 000 injured.^11,12^ To keep the peace in this conflict area, the Royal Thai Army has sent army conscripts to work in the area.^12,13^ and support a transition to peace in the southernmost part of Thailand.^12,14^

The majority of the population in the three southernmost provinces of Thailand is Muslim. The proportion of Muslims among army conscripts is close to that among the general population of approximately 70.9%.^15^ The culture and beliefs of this population may play an important role in sexual behavior.

Studies have been conducted on the sexual behavior of Thai army conscripts.^5,6,8,16,17^ However, to the best of our knowledge, there have been no previous studies on the sexual behavior of Muslim Thai army conscripts in the three southernmost provinces of Thailand. Nevertheless, there are cultural aspects on sexual behavior among Muslim. For example, a study in Ghana suggested that being Muslim were less likely to engage in risky sexual behavior compared to other religion.^18^ The Islamic principle was a deterrent to premarital sexual intercourse.^19,20^ The cultural norms of Islam could potentially prevent social stigma related to HIV infection.^21^ Therefore, in this study, we aimed to explore the sexual behaviors and factors associated with the risk of HIV and other STIs among Muslim army conscripts. The results could be used as baseline information to develop sexual health programs to reduce the risk of HIV and other STIs and to develop sexual health education programs to support sexual health within this population.

## Material and Methods

### Study design and population

This study was conducted using a cross-sectional study design between May and June 2020. The study areas were the three southern border provinces of Thailand: Yala, Pattani, and Narathiwat. These provinces are the border between Thailand and Malaysia with multicultural and multiethnic areas where the majority of the people respect Islam (70.99%).

The study population included Muslim army conscripts from permanent security checkpoints under the regulation of each provincial task force. As the total population could not be accessed in the conflict area for security reasons. The sample size estimation for unknown population was calculated by Cochran’s formula.^22^ (*Note*: n = sample size, e = 0.05, Z = 1.96)

$$n=\frac{Z^{2}}{4e^{2}}=\frac{{\left(1.96\right)}^{2}}{4{\left(0.05\right)}^{2}}=384.16∼\;385$$

That was at least 385 Muslim army conscripts. Purposive sampling was made to select the participants’ workplace, the military operation unit of each province. The security checkpoints under the regulation of provincial military operation were initiate selected from the city center follow by the next farther away security checkpoints of the studied provinces.

The inclusion criterion for the participants of this study was Muslim army conscripts working in the three deep southern provinces of Thailand. The participants had completed their normal military tactic training, had experience in sexual intercourse, and were willing to participate in the study.

### Research tools

The research questionnaire consisted of four parts.

Part I is general information, which consists of age, educational level, marital status, hometown, workplace province, smoking behavior, drinking behavior, substance used, chemical used before sexual intercourse, route of sexual intercourse, number of sexual partners, and history of HIV testing.

Part II is knowledge of the transmission of HIV and other STIs (20 items). Knowledge level was categorized according to Bloom’s cut-off point into three levels^23^: good (≥80%; 16 - 20 points), moderate (60%-79%; 12-15 points), and poor (<60%; <12 points).

Part III is attitude toward condom use (18 items). Attitude level was categorized into three levels according to class interval: good (43-54 points), moderate (31-42 points), and poor attitude (<31 points).

Part IV is sexual behaviors. This part was divided into two groups: high-risk and low-risk HIV/STI. High-risk HIV/STI was defined as having sexual intercourse without condom use at least once. Low-risk HIV/STI was defined as having no sexual intercourse within the past three months, having sexual intercourse with condom use every time, and having sexual intercourse with their wife only without condom use.

### Validity and reliability testing

The questionnaire was tested for content and construct validity by five experts. The index of the item objective congruence (IOC) was used for every part of the questionnaire, including demographic characteristics, knowledge of the transmission of HIV and other STIs, and attitude toward condom use. Each selected item must have at least three experts’ agreement, some items were excised. The IOC score ranged from 0.6 to 1.0. The Cronbach’s α coefficient was used for reliability test for the part of attitude toward condom use. The expert responded to a 3-point scale that consist of 3 (agree), 2 (neutral), and 1 (do not agree). The α coefficient showed good internal consistency (Cronbach’s α coefficient = 0.800). Kuder-Richardson-20 (KR_20_) was used to calculate the dichotomous scale to test the reliability of knowledge on HIV/STI transmission with good internal consistency (KR_20_ coefficient = 0.901). The questionnaire was pilot-tested on 30 Muslim army conscripts from the Songkhla provincial task force.

### Data collection

As the accurate number of the army conscript in the unrest area was not allowed to assess due to security reason; therefore, the excess of 600 questionnaires (200 for each province) were distributed to Muslim army conscripts in order to get the minimum number of respondents. The eligible Muslim army conscripts from each security checkpoint were invited by assigned squad leader to participate in this study. The self-administered questionnaires were completed in a private place, not regulated by the squad leader. Completed response of 421 questionnaires were return which greater than the minimum sample size. However, 61 participants were excluded from the study because of no sexual intercourse experience. Thus, 360 set of questionnaires were included in the analysis which accounted for 93.52% of sample size estimation.

### Data analysis

Descriptive statistics were used to describe the demographic characteristics of the subjects. Univariate analysis was used to test for the association between sexual risk behaviors at the first step. The factors associated with the risk of HIV/STIs behavior with a *P* value less than 0.25 were included for multiple logistic regression.^24^ Correlation analysis was used to test for multicollinearity across variables to avoid the inclusion of highly correlate variables in the same regression model. A forward-stepwise (Likelihood Ratio) method was used to evaluate independent variables associated with sexual risk behaviors that was a dichotomous variable; high-risk of HIV/STIs (coded as 1) and low-risk of HIV/STIs (coded as 0). The statistic gives an odds ratio which controlled for multiple confounders. A *P* value ≤0.05 was considered statistically significant and keep the final model. All statistical analyses were performed using IBM SPSS Statistics for Windows version 25 (IBM Corp., Armonk, NY, USA).

## Results

In total, 360 completed questionnaires were returned to the researcher. Table 1 shows the demographic characteristics, personal behaviors, and sexual risk behaviors of the Muslim army conscripts who participated in this study. Most of them were ≤22 years old (73.9%), which is the age for army recruitment according to law. Most of the participants finished secondary school (62.8%) and were single (73.6%). Aside from the three deep southern provinces, 54% were recruited from other provinces in southern Thailand. Nevertheless, these army conscripts were deployed inthe study areas (i.e., Yala, Pattani, and Narathiwat provinces) in similar proportions.

**Table 1:** Demographic characteristics of the study population (n = 360)

**General informatio**n	**Frequency**	**%**
**Age (mean 20.45 years, max. 26.92 years, min. 20.83 years)**		
22 years and lower	266	73.9
More than 22 years	94	26.1
**Education level**		
Did not study in the education system	7	1.9
Primary school	44	12.2
Secondary school	226	62.8
Vocational study	61	16.9
Bachelor’s degree and above	22	6.1
**Marital status**		
Single	275	76.4
Married	85	23.6
**Hometown**		
Three southern provinces	165	45.8
Other provinces	195	54.2
**Deployment area**		
Yala province	128	35.6
Pattani province	126	35.0
Narathiwat province	106	29.4
**Smoking behavior**		
Do not smoke/quit smoking	158	43.9
Smoke	202	56.1
**Drinking behavior**		
Do not drink/Had been drunk	208	57.8
Drink	152	42.2
**Substance used**		
Do not use/stopped using	350	97.2
Substance use	10	2.8
**Chemical used before sexual intercourse (n=119)** ^*^		
Alcohol	51	42.9
Cigarette	62	52.1
Substance	10	8.4
Stimulant	22	18.5
**Sexual intercourse**		
Had no sexual intercourse within the last three months^a^	97	26.9
Single (65, 67.0%), married (32, 33%)		
Had sexual intercourse^*^	263	73.1
Vaginal sexual intercourse	248	94.3
Oral sexual intercourse	64	24.3
Anal sexual intercourse	24	9.1
**Number of sexual partners (n=263)**		
One partner	176	66.9
Married^a^	53	30.1
Single	123	69.9
Use condoms every time^a^	38	30.9
Inconsistent condom use^b^	39	31.7
Never used condoms^b^	46	37.4
More than one partner	87	33.1
Use condoms every time^a^	35	40.2
Inconsistent condom use^b^	37	42.5
Never used condoms^b^	15	17.2
**History of HIV testing**		
No	229	63.6
Yes	131	36.4

^*^ Some participants used more than one route. ^a^ Low-risk HIV/STI (223 participants, 61.9%). ^b^ High-risk HIV/STI (137 participants, 38.1%).

Regarding personal behavior, most of the participants were active smokers (56.1%) and drinkers (42%) and reported no substance use (97.2%). The participants reported their alcohol consumption (14.2%), smoking behavior (17.2%), substance use (2.8%), and stimulant use (6.1%) before sexual intercourse. They also reported other routes of intercourse, namely, oral (24.3%) and anal (9.1%), other than vaginal sexual intercourse (93.9%) during the last three months. About 67% had one sexual partner. Most of the participants were never screened for HIV testing (63.6%). The details are listed in Table 1.

**Figure 1:**
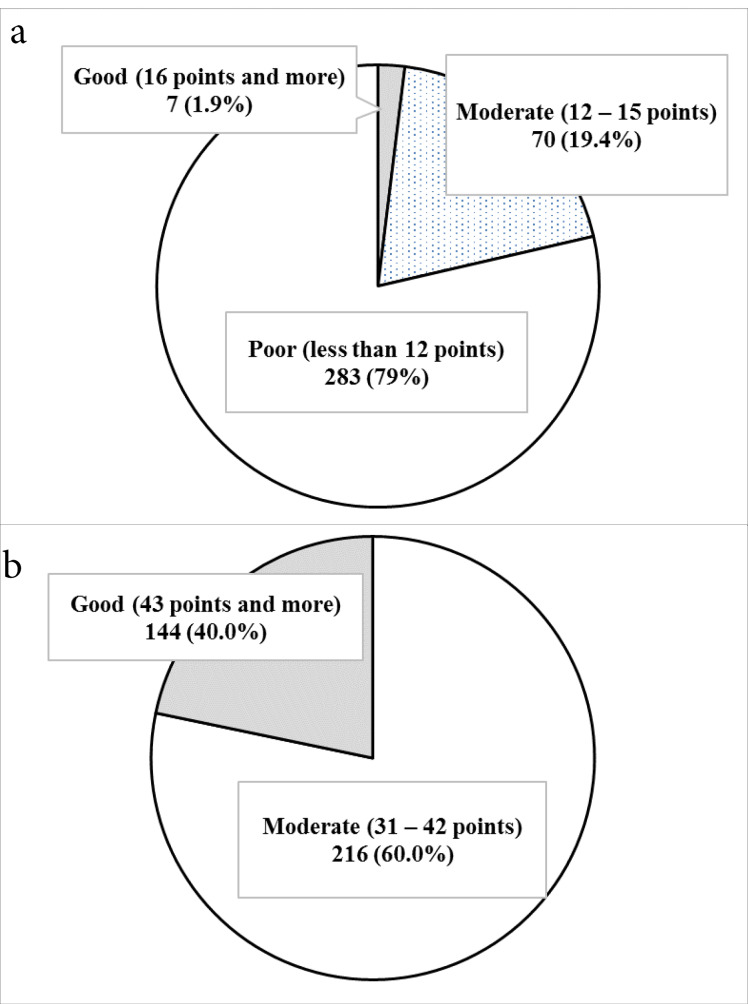
a). Knowledge on HIV/STIs transmission b). Attitude toward condom use.

Figure 1 shows the knowledge levels of the participants and the attitude toward the condom use, most of the army conscripts had a poor level of knowledge on HIV/STI transmission (78.7%), and their knowledge was at a good level (1.9%). Among these, 60% of the participants had a moderate attitude level, while 40% had a high attitude level toward the condom use.

Table 2 shows that some participants correctly answered the true/false statements. However, many responses indicated uncertainty and a lack of knowledge. Most participants knew that sharing needles during intravenous injection could spread HIV (73.6%), that HIV could be transmitted through blood transfusions (68.6%), and that HIV testing could encourage safer sex practices (55.8%). The majority also correctly identified the following statements as false: well-educated people have no risk of HIV (56.1%), there is no risk of HIV if they have sex with a student (54.7%), and they will have no risk of HIV if they wipe other men’s semen out of the vagina or anus before having sexual intercourse (47.8%). A significant number of participants did not know that HIV could not be prevented or cured by antibiotics (47.0%), that there is no vaccine for HIV (43.3%), or that they could be at risk for HIV infection even if they used lubricant gel (41.7%).

**Table 2:** Knowledge of the transmission of HIV and other STIs

**Statement**	**Frequency (%)**		
	**True**	**False**	**Not sure**
1. Withdrawing the penis to ejaculate outside of the vagina/anal/oral area can protect you from HIV and other STIs*	135 (37.5)	131 (36.4)	94 (26.1)
2. HIV can be contracted through blood transfusion	247 (68.6)	55 (15.3)	58 (16.1)
3. HIV can be transmitted by kissing*	121 (33.6)	131 (36.4)	108 (30.0)
4. HIV is spread only by sex workers*	111 (30.8)	165 (45.8)	84 (23.4)
5. HIV can be spread by sharing needles during intravenous drug use	265 (73.6)	43 (12.0)	52 (14.4)
6. Coughing and sneezing do not spread HIV	145 (40.3)	106 (29.4)	109 (30.3)
7. A person can get HIV by sharing a glass of water with someone who has HIV*	98 (27.2)	138 (38.3)	124 (34.5)
8. Showering, or washing one’s genitals/private parts, after sex keeps a person from getting HIV*	118 (32.8)	129 (35.8)	113 (31.4)
9. People who have been infected with HIV quickly show serious signs after being infected*	99 (27.5)	125 (34.7)	136 (37.8)
10. There is a vaccine that can stop people from getting HIV*	99 (27.5)	105 (29.2)	156 (43.3)
11. A person will not get HIV if he or she is taking antibiotics*	85 (23.6)	106 (29.4)	169 (47.0)
12. Having sex with more than one partner can increase a person’s chances of being infected with HIV and other STIs	198 (55.0)	80 (22.2)	82 (22.8)
13. A person can get HIV by using the same swimming pool as a person who has HIV*	77 (21.4)	164 (45.5)	119 (33.1)
14. A person can get HIV from oral sex	117 (32.5)	96 (26.7)	147 (40.8)
15. Using lubricant gel while having sexual intercourse eliminates the risk of getting HIV*	72 (20.0)	138 (38.3)	150 (41.7)
16. HIV can be transmitted by a mosquito bite*	93 (25.8)	154 (42.8)	113 (31.4)
17. There is no risk of HIV if you have sexual intercourse with a student*	74 (20.6)	197 (54.7)	89 (24.7)
18. Wiping other men’s semen out of the vagina or anus can protect you from HIV *	84 (23.3)	172 (47.8)	104 (28.9)
19. Having sex with highly educated partners poses no risk of HIV*	84 (23.3)	202 (56.1)	74 (20.6)
20. HIV testing can encourage sexual behavior that protects against HIV	201 (55.8)	63 (17.5)	96 (26.7)

*False statements.

Table 3 shows detailed information about attitudes toward condom use. The majority of the participants agreed that HIV/STI could be prevented by using condoms (86.9%), that both they and their sexual partners could be protected from sexually transmitted diseases when they use condoms (74.7%), and that using condoms every time they have sexual intercourse could prevent the risk of HIV/STIs (71.7%).

**Table 3:** Attitude toward condom use

**Statement**	**Attitude, n (%)**		
	**Agree**	**Undecided**	**Disagree**
1. Condom use may help prevent HIV/STIs	313 (86.9)	36 (10.0)	11 (3.1)
2. I feel embarrassed when buying condoms*	108 (30.0)	164 (45.6)	88 (24.4)
3. I do not like using condoms because of the unpleasant smell*	91 (25.3)	152 (42.2)	117 (32.5)
4. The proper use of condoms increases mental pleasure during sex	211 (58.6)	126 (35.0)	23 (6.4)
5. Condom use makes me feel sexual pleasure	105 (29.2)	161 (44.7)	94 (26.1)
6. Condoms are not needed when having sexual intercourse with students*	92 (25.6)	79 (21.9)	189 (52.5)
7. Condoms must be used even when having sexual intercourse with good-looking and nice partners	206 (57.2)	115 (32.0)	39 (10.8)
8. I do not like using condoms because they are sticky and oily*	113 (31.4)	140 (38.9)	107 (29.7)
9. Condom use is a religious taboo*	95 (26.4)	97 (26.9)	168 (46.7)
10. It’s normal to buy condoms at a convenient or drug store	218 (60.5)	123 (34.2)	19 (5.3)
11. Condom use can protect you and your sexual partner	269 (74.7)	75 (20.8)	16 (4.5)
12. I do not like using condom when my sexual partner gives me oral sex*	138 (38.4)	129 (35.8)	93 (25.8)
13. It’s normal to carry condoms and be ready to use them when having sexual intercourse	210 (58.3)	104 (28.9)	46 (12.8)
14. Using condoms every time during sexual intercourse can prevent the risk of HIV/STIs	258 (71.7)	80 (22.2)	22 (6.1)
15. Using condoms in the proper size that fits your penis can prevent HIV/STIs	214 (59.5)	112 (31.1)	34 (9.4)
16. After ejaculation, you should remove the condom while your penis is still erect	191 (53.1)	129 (35.8)	40 (11.1)
17. Using two pieces of condoms during sexual intercourse may enhance their effectiveness to prevent HIV/STIs*	109 (30.3)	106 (29.4)	145 (40.3)
18. I prefer my sexual partner to use condoms or dental dams before oral sex	138 (26.4)	127 (35.2)	95 (26.4)

*Negative statements

Table 4 shows the association between the studied factors and the risk of sexual behavior. For multiple logistic regression (Table 5),the results suggest that marital status, chemical used before sexual intercourse, and route of sexual intercourse were associated with the risk of HIV/STI behavior (*P*< 0.05).

**Table 4:** Univariate analysis between the studied factors and risk of HIV/STIs

**Factors**	**Odds Ratio**	**95% CI for EXP (B)**	
		**Lower**	**Upper**
Age (21-22 vs. > 22 year)	1.145	0.707	1.852
Education level	0.247		
Did not study in the education system	0.625	0.112	3.477
Primary school	0.761	0.273	2.124
Secondary school	0.466*	0.193	1.125
Vocational study	0.407*	0.150	1.099
Bachelor’s degree and above	[Ref]		
Marital status (married vs. single [ref])	0.197*	0.102	0.380
Hometown (three southern provinces vs. others)	1.038	0.677	1.592
Deployment area	0.247		
Yala province	1.512*	0.888	2.576
Pattani province	1.080	0.628	1.858
Narathiwat province	[Ref.]		
Smoking behavior (yes vs. no [ref])	1.898*	1.223	2.946
Drinking behavior (yes vs. no [ref])	1.345*	0.875	2.067
Substance used (yes vs. no [ref])	0.398*	0.083	1.903
Chemical used before sexual intercourse			
Alcohol (yes vs. no [ref])	2.453*	1.344	4.477
Cigarette (yes vs. no [ref])	2.299*	1.321	4.001
Substance (yes vs. no [ref])	3.949*	1.004	15.536
Stimulate (yes vs. no [ref])	3.059*	1.248	7.497
Route of sexual intercourse (n = 263)			
Vaginal (yes vs. no [ref])	19.781*	8.375	46.719
Oral (yes vs. no [ref])	4.802*	2.681	8.601
Anal (yes vs. no [ref])	2.923*	1.242	6.880
Knowledge level on HIV/STI transmission	0.597		
Good (16 points and more)	[ref]		
Moderate (12–15 points)	1.162	0.255	5.292
Poor (less than 12 points)	0.758	0.436	1.318
Attitude level toward condom use	0.645*	0.414	1.003
(Good vs. moderate [ref])			
Number of Sexual partners (n = 263)	4.562*	3.108	6.695
(> 1 vs 1 partner [ref])			
History of HIV testing (yes vs. no [ref])	1.115	.718	1.733

^a^ Fisher’s exact test. * *P* < 0.25.

**Table 5:** Association of predicted variables and risk of HIV/STIs

**Variables**	**Odds ratio**	**95% CI for EXP (B)**		***P *** **value**
		**Lower**	**Upper**	
Marital status (married vs. single [ref])	0.078	0.035	0.172	< 0.001
Chemical used before sexual intercourse				
Substance (yes vs. no [ref])	8.044	1.288	50.230	0.026
Stimulate (yes vs. no [ref])	3.632	1.080	12.211	0.037
Route of sexual intercourse				
Vaginal (yes vs. no [ref])	26.228	8.370	82.189	< 0.001
Oral (yes vs. no [ref])	2.256	1.106	4.601	0.025
Constant				

-2 Log likelihood 304.495, Cox & Snell R square 0.383, Nagelkerke R square 0.521.

## Discussion

HIV/STIs are sexually transmitted diseases that cause a serious burden on patients and the healthcare system. To reduce the number of new cases, good knowledge, attitudes, and practices are necessary. However, these important elements are still limited in some groups of the population. In this study, we found that the level of knowledge on HIV/STIs among Muslim army conscripts was poor (78.7%). Although most participants finished secondary school (62.8%), through which they had already received basic sexual health education, their responses show that they have limited knowledge. Based on their responses, the lower scores were due to their limited basic knowledge. Most of them could not decide whether the statements were correct, leading to a low score for each item.

Among the 360 Muslim army conscripts, 67.0% single participants abstained from sex within the last three months (Table 1). This group of participants obeyed the Islamic principle that disallows sexual intercourse outside of marriage. Sexual abstinence is a means of self-protection from HIV/STIs.^25,26^ About 18.3% of the participants were married and had no sexual intercourse other than with their wives, and 4.2% had 2-4 sexual partners other than their wives. This result indicates that 50.6% of the Muslim army conscripts had premarital and/or extramarital sexual intercourse, consistent with other studies on the Muslim population.^20^

Half of the Muslim army conscripts (50.6%) had sexual intercourse outside of marriage, but the study found that most of them had no history of HIV testing. Although one study indicated a negative relationship between HIV prevalence and being Muslim,^27^ this study was conducted way back in 2004. A recent study showed that the HIV prevalence rate in Muslim-majority countries was increasing.^28^ The result on sexual intercourse outside of marriage is consistent with a study in Malaysia that found that among 90% of Muslim participants, 35.6% had a lifetime sexual partner, and 64.4% had more than one sexual partner.^29^ Nevertheless, an inconsistent finding related to sexual intercourse outside of marriage was also observed among sexually active Muslim across countries. A study conducted in USA found that more than half of US Muslim college students reported ever having had sexual intercourse,^30^ while approximately 27.5% Muslim men in Ghana involved in high-risk sexual behavior.^18^ In Thailand, a study in the southern border of Thailand (Muslim majority community) found that 9.0% schools and a vocational college students had experience of sexual intercourse^31^ and 4.0% Muslim adolescent men in *Ponok* (private Islamic school) in Southern Thailand had premarital sexual intercourse.^20^ The differences of the number observed among these studies may result from the local cultural influencing. Muslim in western countries or non-Muslim community may influence by their peers, media, local culture, and living environment. These factors potentially affect their belief, religious education, and lifestyle which also including their sexual behavior. However, we did not conduct in depth interview among the participants in our study to find the actual reason for this result.

Our study found that marital status was associated with sexual risk behaviors, with single Muslim army conscripts having more sexual risk behaviors than the married participants. This finding is similar to a study on HIV infection and related risk factors among new Thai army conscripts, in which being single was found to be a risk factor associated with HIV infection.^6^ Single males have a freer lifestyle than married men, and they have more chances of having sexual contact and more sexual partners. This may lead them to be more at risk of HIV/STIs. A study on Thai army conscripts confirmed that HIV-positive males were more likely to have had sex with another man, have more reported sexual contact with female sex workers, and have had more lifetime sexual partners.^32^ Our findings are also comparable with those of studies conducted in Poland and the United States, which reported that having sexual contact with sex workers, casual sex with strangers, sex with multiple partners, and not using condoms during sexual intercourse were associated with HIV/STIs.^33,34^ Moreover, another study suggested that having sexual intercourse with men, civilian partners, and unprotected anal sexual intercourse increased HIV/STIs among army.^35^ Unprotected sexual intercourse seems to be a major risk, as this factor was also reported in a study, which indicated that only 12% of sexually active males used condoms during sexual intercourse.^36^

In our study, personal behaviors (i.e., substance and stimulant use before sexual intercourse) were also found to be significantly associated with sexual risk behaviors. A similar result was observed in a study on an HIV surveillance program for military conscripts in Thailand, which found substance use to be a major risk factor for HIV.^17^ Studies conducted in Malaysia and Iraq also found an increasing rate of HIV prevalence among Muslim populations due to shared injection equipment.^29,37^

Although our study successfully assessed the level of knowledge and associated risk factors for HIV/STIs, several limitations need to be addressed. First, we were unable to obtain the total number of Muslim army conscripts from each province because of security reasons. Thus, we could not determine the sample size of each province. Nevertheless, we tried to send out as many questionnaires as possible to reach our expected sample size. Second, the questionnaire used in this study was not a standard questionnaire, but it was constructed by researcher team. However, it was evaluated by 5 public health experts, as well as Muslim academicians in the study area. Third, the data collection process could not be conducted in red areas, which are the most dangerous zones of unrest in the three deep southern provinces of Thailand. Thus, the participants could not represent the entire population in each province. Lastly, the Muslim army conscripts who participated were recruited from the security checkpoint in the city center and then circularly spread out. Their risky behaviors could potentially be higher farther the deployment stations, as they would be closer to amusement places and other entertainment venues. This could have overestimated the risky behaviors of all Muslim army conscripts in all the study areas.

## Conclusion

This study found that the knowledge level on HIV/STI transmission of Muslim army conscripts was poor, and that attitude toward condom use was at a moderate level. The participants’ characteristics and behaviors were found to be important and associated with sexual risk behaviors. To increase the knowledge and understanding of Muslim army conscripts, sufficient communication and clarification about HIV/STI transmission should be made. In addition, programs on sexual health and risky behavior prevention or sexual health education should be developed according to Islamic principles. Programs for lowering the risk of HIV/STIs according to religious doctrines should also be implemented.

## Acknowledgements

It is appreciated all experts for valuable suggestion and evaluation. Thanks to commander in chiefs for permission to collect the data and helpful squad leaders as well as all army conscripts.

## Funding

Not applicable.

## Competing interests

The authors declare that there is no conflict of interests.

## Ethical approval

The participants were verbally invited to participate in the study. They were exempted from signing the informed consent forms because some questions were about illegal behaviors and/or religious taboo or sin. Ethical consideration was approved by the Ethics Review Committee for Research Involving Human Research Subjects, Health Science Group, Chulalongkorn University (Project number 271.1/62) and the Ethics Committee for Human Research Subjects of Sirindhorn College of Public Health, Yala (Project number: 026–63) as the local ethics committee of the study area.

## Authors’ contributions

AS was involved in the literature search, design, and data collection. KY was a consultant for human ethic research especially in vulnerable participants. KY and KK gave critically suggestion and recommendation for data analysis, interpretation and draft proving. KK made concise appraisal and approved final version before publishing. All authors have agreement to be accountable for all aspects of the work.

## References

[1] National AIDS Committee. Thailand National Strategy to End AIDS 2017-2030. Bangkok: N.C. Concept Co. Ltd; 2017.

[2] UNAIDS. HIV and AIDS Estimates: Country fact sheet 2019. Available from: https://www.unaids.org/en/regionscountries/countries/thailand. Accessed 13 August 2020.

[3] Stahlman S, Javanbakht M, Cochran S, Hamilton AB, Shoptaw S, Gorbach PM (2014). Self-reported sexually transmitted infections and sexual risk behaviors in the U.S. military: how sex influences risk. Sex Transm Dis.

[4] Julawong O, Srikanok W (2013). Development of HIV prevention motivation model for promoting healthy behavioral changes in conscripts of the ninth of battalion infantry. J Nurs Educ Pract.

[5] Surit P, Jariya W, Zheng N, Yi H, Yu X, Srithong W (2017). Risk factors affecting condom use among Royal Thai Army conscripts in Thailand. World J AIDS.

[6] Jose JE, Sakboonyarat B, Kana K, Chuenchitra T, Sunantarod A, Meesiri S (2020). Prevalence of HIV infection and related risk factors among young Thai men between 2010 and 2011. PLoS One.

[7] Amnesty International. Physical, mental, and sexual abuse of conscripts in Thailand’s military. London, UK: Amnesty International Ltd; 2020.

[8] Saengdidtha B, Rangsin R, Kaoaiem H, Sathityudhakarn O (2016). Risk factors for HIV infection among Thai young men aged 21-23 years. Epidemiology (Sunnyvale).

[9] Biague A, Månsson F, da Silva Z, Dias F, Nantote Q, Costa J (2010). High sexual risk taking and diverging trends of HIV-1 and HIV-2 in the military of Guinea-Bissau. J Infect Dev Ctries.

[10] Saengdidtha B, Rangsin R, Kana K, Kaoaiem H (2012). [The uses of epidemiologic and public health approaches for HIV/AIDS control among young men in the Royal Thai Army and Thailand]. Sanid Mil.

[11] Parks T, Colletta N, Oppenheim B. The Contested Corners of Asia: Subnational Conflict and International Development Assistance. The Case of Southern Thailand. The Asia Foundation; 2013. Available from: https://asiafoundation.org/wp-content/uploads/2013/10/The-Contested-Corners-of-Asia_Subnational-Conflict-and-International-Development-Assistance.pdf. Accessed 10 September 2020.

[12] The division of operational strategic management of south border. Operational Strategic Management of South Border Provinces (2014-2017). 2017. Yala: Thailand. Available from: http://www.planning.psu.ac.th/index.php/news/53-south-border. Accessed 24 July 2020.

[13] Smith AL (2004). Trouble in Thailand’s Muslim South: separatism, not global terrorism. Asia Pacific Center for Security Studies.

[14] Klein J. Democracy and Conflict in Southern Thailand: A Survey of the Thai Electorate in Yala, Narathiwas, and Pattani. Thailand: The Asia Foundation; 2010. Available from: http://patricklepetit.jalbum.net/PATTANI/LIBRARY/Democracy%20and%20conflict.pdf. Accessed 6 April 2019.

[15] Yala provincial office. Yala Developmental Plan (2018-2022) Revised edition. Available from: http://www.yala.go.th/content/plan/2561-2565/plan2564.pdf. Accessed 24 July 2021.

[16] Jose JE, Sakboonyarat B, Mungthin M, Nelson KE, Rangsin R (2021). Rising prevalence of HIV infection and associated risk factors among young Thai men in 2018. Sci Rep.

[17] Nelson KE, Rangsin R (2017). The importance of military conscripts for surveillance of human immunodeficiency virus infection and risk behavior in Thailand. Curr HIV Res.

[18] Gyimah SO, Tenkorang EY, Takyi BK, Adjei J, Fosu G (2010). Religion, HIV/AIDS and sexual risk-taking among men in Ghana. J Biosoc Sci.

[19] Ghaffari M, Gharlipour Gharghani Z, Mehrabi Y, Ramezankhani A, Movahed M (2016). Premarital Sexual Intercourse-Related Individual Factors Among Iranian Adolescents: A Qualitative Study. Iran Red Crescent Med J.

[20] Sateemae S, Abdel-Monem T, Sateemae M (2015). Religiosity and social problems among Muslim adolescents in southern Thailand. J Muslim Ment Health.

[21] Hasnain M (2005). Cultural approach to HIV/AIDS harm reduction in Muslim countries. Harm Reduct J.

[22] Cochran WG. Sampling Techniques. Hoboken: John Wiley & Sons; 2007.

[23] Bloom BS (1968). Learning for Mastery. Evaluation Comment.

[24] Bendel RB, Afifi AA (1977). Comparison of stopping rules in forward “stepwise” regression. J Am Stat Assoc.

[25] Piriyasart J, Songwathana P, Kools S (2018). Perceptions of sexual abstinence among Muslim adolescent girls in southern Thailand. Int J Adolesc Med Health.

[26] Sudan SA (2015). Educating children on sexual matters based on the teaching of Islam: the role of Muslim parents. J Educ Soc Policy.

[27] Gray PB (2004). HIV and Islam: is HIV prevalence lower among Muslims?. Soc Sci Med.

[28] Yamaguchi K. HIV/AIDS in the Muslim-Majority Countries: Formula for Low Prevalence. Bemidji State University: [cited 2020 Jul 9]. Available from: https://www.bemidjistate.edu/academics/departments/political-science/wp-content/uploads/sites/40/2015/05/kaoru-yamaguchi_thesis.pdf.

[29] Nik Farid ND, Rus SC, Dahlui M, Al-Sadat N, Aziz NA (2014). Predictors of sexual risk behaviour among adolescents from welfare institutions in Malaysia: a cross sectional study. BMC Public Health.

[30] Ahmed S, Abu-Ras W, Arfken CL (2014). Prevalence of risk behaviors among U.S. Muslim college students. J Muslim Ment Health.

[31] Hayee F, Fongkaew W, Chanprasit C, Kaewthummanukul T, Voss JG. Sexual risk behaviors and influencing factors among Muslim adolescents on southern border of Thailand. Int J Adolesc Med Health. 2020. 10.1515/ijamh-2019-0221 32549162

[32] Rangsin R, Kana K, Chuenchitra T, Sunantarod A, Mungthin M, Meesiri S (2015). Risk factors for HIV infection among young Thai men during 2005-2009. PLoS One.

[33] Stahlman SL. Sexually Transmitted Infections in the U.S. Military: How Gender Influences Risk [dissertation]. Los Angeles: University of California; 2014.

[34] Korzeniewski K. Sexually transmitted infections among army personnel in the military environment. In: Malla N, ed. Sexually Transmitted Infections. Rijeka, Croatia: IntechOpen; 2012. 10.5772/31245

[35] Hakre S, Scoville SL, Pacha LA, Peel SA, Kim JH, Michael NL (2015). Brief report: sexual risk behaviors of HIV seroconverters in the US army, 2012-2014. J Acquir Immune Defic Syndr.

[36] Ismael AS, Sabir Zangana JM (2012). Knowledge, attitudes and practice of condom use among males aged (15-49) years in Erbil Governorate. Glob J Health Sci.

[37] Loue S (2011). AIDS jihad: integrating the Islamic concept of jihad with HIV prevention theory. J Health Care Poor Underserved.

